# Interactomic analysis of REST/NRSF and implications of its functional links with the transcription suppressor TRIM28 during neuronal differentiation

**DOI:** 10.1038/srep39049

**Published:** 2016-12-15

**Authors:** Namgyu Lee, Sung Jin Park, Ghazal Haddad, Dae-Kyum Kim, Seon-Min Park, Sang Ki Park, Kwan Yong Choi

**Affiliations:** 1 Department of Life Sciences, Pohang University of Science and Technology, Pohang, Gyeongbuk, Korea; 2 Department of Molecular Genetics, University of Toronto, Toronto, Ontario M5S 3E1, Canada; 3 Donnelly Centre, Departments of Molecular Genetics and Computer Science, University of Toronto, Toronto, Ontario M5S 3E1, Canada; 4 Lunenfeld-Tanenbaum Research Institute, Mount Sinai Hospital, Toronto, Ontario M5G 1X5, Canada; 5 Pohang Center for Evaluation of Biomaterials, Pohang, Gyeonbuk, Korea; 6 Department of Veterinary Medicine, Kyungpook, National University, Daegu, Republic of Korea; 7 Department of Integrative Biosciences & Biotechnology, Pohang, Gyeongbuk, Republic of Korea

## Abstract

RE-1 silencing transcription factor (REST) is a transcriptional repressor that regulates gene expression by binding to repressor element 1. However, despite its critical function in physiology, little is known about its interaction proteins. Here we identified 204 REST-interacting proteins using affinity purification and mass spectrometry. The interactome included proteins associated with mRNA processing/splicing, chromatin organization, and transcription. The interactions of these REST-interacting proteins, which included TRIM28, were confirmed by co-immunoprecipitation and immunocytochemistry, respectively. Gene Ontology (GO) analysis revealed that neuronal differentiation-related GO terms were enriched among target genes that were co-regulated by REST and TRIM28, while the level of CTNND2 was increased by the knockdown of REST and TRIM28. Consistently, the level of CTNND2 increased while those of REST and TRIM28 decreased during neuronal differentiation in the primary neurons, suggesting that CTNND2 expression may be co-regulated by both. Furthermore, neurite outgrowth was increased by depletion of REST or TRIM28, implying that reduction of both REST and TRIM28 could promote neuronal differentiation via induction of CTNND2 expression. In conclusion, our study of REST reveals novel interacting proteins which could be a valuable resource for investigating unidentified functions of REST and also suggested functional links between REST and TRIM28 during neuronal development.

RE-1 silencing transcription factor (REST), which is also known as neuron-restrictive silencer factor (NRSF), is a transcription repressor that binds to the 21-bp RE1 sites in the regulatory regions of its target genes^1^. REST is known to have a central role in regulating neurogenesis, neural differentiation, and preservation of the unique neural phenotype^2^. Downregulation of REST during neural differentiation is necessary for the correct development of certain classes of neurons^3^. REST levels are downregulated by proteasomal degradation when embryonic stem cells differentiate into neural stem cell and decrease by transcriptional repression during the differentiation of neural progenitor cells^2^,^4^. REST and its target genes have also been implicated in the pathogenesis and therapeutic mechanism of neurodegenerative diseases, such as schizophrenia, ischemic strokes, Huntington disease, epilepsy, Alzheimer’s disease, Parkinson’s disease, and mood disorders^2^. Therefore, REST-interacting proteins need to be identified to better understand the functions mediated by this transcription factor.

REST is known to repress its target genes by interacting with subunits of several transcription regulatory complexes, including CoREST and mSin3 corepressor complexes, the SWItch/Sucrose Non-Fermentable (SWI/SNF) complex, and polycomb repressive complex 1 (PRC1) and PRC2^1^,^5^,^6^. These REST-interacting proteins were independently identified using yeast two-hybrid screening or co-immunoprecipitation under different experimental conditions. However, a systematic global analysis of the REST interactome has not yet been performed. Following recent advancements in mass spectrometry, interactomic studies using mass spectrometry-based proteomics are being widely used for the systematic identification of binding proteins in a relatively unbiased manner^7^, with the combination of affinity purification and mass spectrometry analysis (AP–MS) in particular emerging as a powerful strategy for characterizing protein interactions^7^.

Tripartite motif-containing 28 (TRIM28), which is also known as KRAB-associated protein-1, is a scaffold protein involved in transcriptional regulation, playing a major role as a corepressor in many repression complexes^8^. TRIM28 binds to the conserved Krüppel-associated box zinc finger (KRAB) repression domain of many transcription factors, and the resulting TRIM28-associated transcription complexes have been implicated in multiple aspects of cellular physiology, including genome stability, immune responses, prevention of virus integration, and early embryonic development^8^,^9^. TRIM28 has also been shown to promote pluripotency in embryonic stem cells through the repression of differentiation-inducible genes and the depression of pluripotency-associated genes^10^. However, although several TRIM28-associated transcription factors have been identified, such as hetero-chromatin-associated protein 1, nuclear corepressor, histone deacetylases, and histone methyltransferases, the identification of additional TRIM28-interacting transcription factors could help in elucidating how this protein regulates gene expression under specific conditions^9^.

In this study, we identified 204 REST-interacting proteins using AP–MS and carried out a systematic analysis of their interactome. Of these proteins, the nuclear and cytoplasmic proteins were mostly enriched, reflecting the nuclear and cytoplasmic localization of REST. The interaction networks of REST indicated its involvement in biological processes associated with mRNA processing, chromatin organization, and transcription. The interactions and co-localizations of eight candidate REST-interacting proteins were confirmed by co-immunoprecipitation and immunocytochemistry, respectively. Based on a public microarray database, we confirmed that a significant number of genes are co-regulated by REST and TRIM28, of which CTNND2 was found to have the highest cluster coefficient using the network analysis. We confirmed that the knockdown of REST or TRIM28 increased the level of CTNND2 in SH-SY5Y cells and that the mRNA level of CTNND2 gradually increased as REST and TRIM28 decreased during differentiation of the primary neurons. Furthermore, neurite outgrowth was increased by knockdown of REST or TRIM28, suggesting that downregulation of both REST and TRIM28 might promote neuronal differentiation via induction of CTNND2 expression. Thus, this interactomic study revealed novel interacting proteins that may represent a valuable resource for investigating additional functions of REST and also suggested functional links between REST and TRIM28 during neuronal development.

## Results

### Identification of REST-interacting proteins by LC–MS/MS

**Figure 1:**
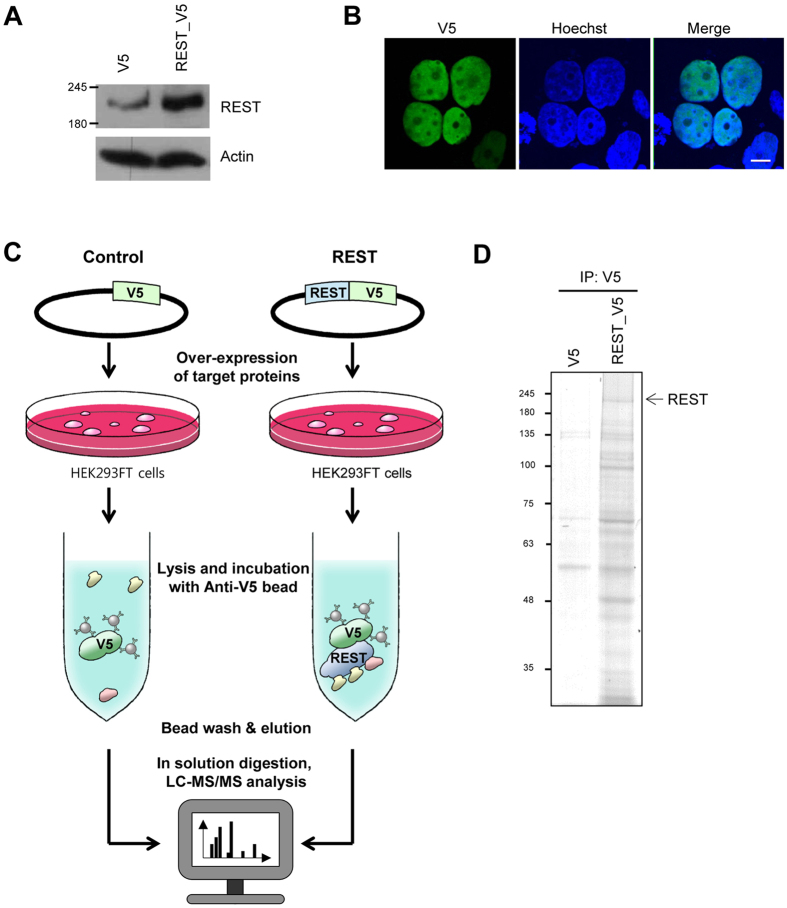
Experimental scheme and analysis of REST-interacting proteins. (**A**) Western blot analysis of REST and actin. HEK293FT cells were transfected with control and REST-V5 expressing vector, respectively. Levels of REST and actin were assessed by western blotting. (**B**) Immunofluorescence staining to confirm localization of transiently expressed REST. HEK293FT cells transfected with the REST-V5 expressing vector were fixed after 24 h, followed by probing with V5 antibody. Images analyzed at magnification of 400x. Scale bar indicates 10 μm. (**C**) Experimental procedures for LC-MS/MS analysis after immunoprecipitation of REST. V5-tagged REST proteins were expressed in HEK293FT cells and immunoisolated using anti-V5 antibody immobilized on agarose beads. Immunocomplexes were eluted and digested for LC-MS/MS analysis. (**D**) SDS-PAGE and Coomassie blue staining of immunocomplexes. The arrow indicates REST. Full-length gels and blots are included in the Supplementary Figure 3.

We confirmed the efficiency of the REST expression vector by western blot analysis of cells transfected with a previously constructed V5-conjugated REST expression vector (Fig. 1A 11). The V5-REST band was located at around 200 kD on the gel and utilized for the further analysis. Since artificial tagging can sometimes cause proteins to mislocalize in cells, we also checked the localization of V5-tagged REST proteins using immunofluorescence staining, which showed that exogenous REST proteins were mainly localized in the nucleus, as previously reported (Fig. 1B 12). The V5-conjugated REST expression vector was transiently transfected into HEK293FT cells, which have been widely used to isolate interacting proteins^13^,^14^,^15^,^16^. The V5-tagged REST proteins were expressed and immunoisolated using an anti-V5 antibody immobilized on agarose beads. We also identified the REST-interacting proteins using in-solution digestion^17^,^18^ followed by triplicate LC–MS/MS analyses using an LTQ Orbitrap Velos™ mass spectrometer (Fig. 1C). Candidate REST-interacting partners were identified via a database search using the MaxQuant software followed by label-free quantification. Enrichment of the REST proteins with interacting proteins during co-immunoprecipitation was confirmed using SDS-PAGE analysis, and the efficient elution of REST and co-isolated proteins from the agarose beads was verified (Fig. 1D).

**Figure 2:**
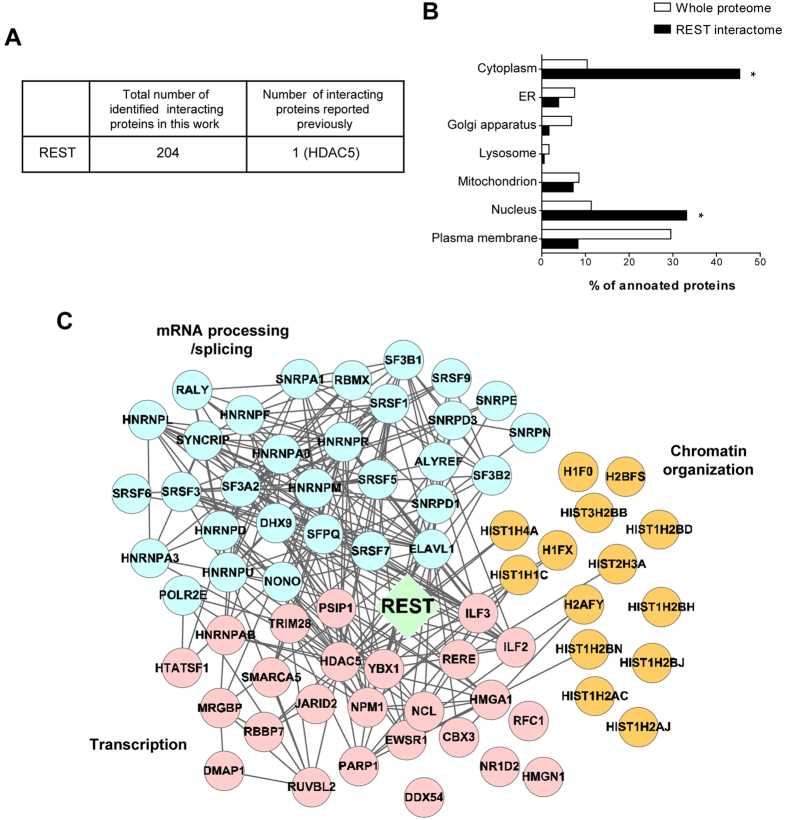
Systematic analysis of REST interactome. (**A**) Comparison of our REST interactome with REST-interacting proteins reported previously. (**B**) Subcellular localizations of REST interactome. (**C**) A network model delineating the relationship between REST-interacting proteins. The node color indicates the functional module of the interacting partners; mRNA processing/splicing (blue), transcription (pink) and chromatin organization (orange). Edges were drawn based on the public protein-protein interaction database (gray). Fisher’s test was used for statistical analysis. (*P < 0.05).

We identified 237 REST-interacting proteins (Supplementary Table 1) based on a total of 1214 unique peptides (Supplementary Table 2). Candidate proteins with more than two unique peptides and a posterior error probability (PEP) < 0.01 were selected for further analysis, and all reverse sequences and contaminant proteins were removed. Overall, 204 interacting partners were enriched in the REST-positive sets (Supplementary Table 3), including histone deacetylase 5 (HDAC5), which had previously been identified in seven different databases (Fig. 2A 19,20,21,22,23,24,25).

### Systematic analysis of the REST interactome

To verify the biological relevance of the identified interactomic proteins, several properties of the proteome were examined. We found that nuclear and cytoplasmic proteins were enriched (*P* < 0.05; Fig. 2B). A functional enrichment analysis and subsequent interactomic analysis of REST indicated various functions, including mRNA processing/splicing, chromatin organization, and transcription. To identify the proteins corresponding to each specific function, we reconstructed a network model utilizing REST-interacting proteins that are involved in these processes (Fig. 2C). This interactome included some histone proteins involved in chromatin organization, such as histone H4 (HIST1H4A), core histone macro-H2A.1 (H2AFY), and histone H2A type 1-C (HIST12AC) (Fig. 2C). We also identified 24 novel REST-interacting proteins involved in transcription, including nucleophosmin1 (NPM1), nucleolin (NCL), poly[ADP-ribose]polymerase1 (PARP1), TRIM28, and high mobility group protein HMG-I/HMG-Y (HMGA1) (Fig. 2C). The functional network analysis also identified 31 novel interacting proteins related to mRNA processing/splicing, such as THO complex subunit 4 (ALYREF) and heterogeneous nuclear ribonucleoprotein (HnRNP) family proteins. Accordingly, seven REST-interacting protein candidates were subjected to further analysis to validate and characterize their interactions with REST.

### Confirmation of interactions between interacting proteins and REST

**Figure 3:**
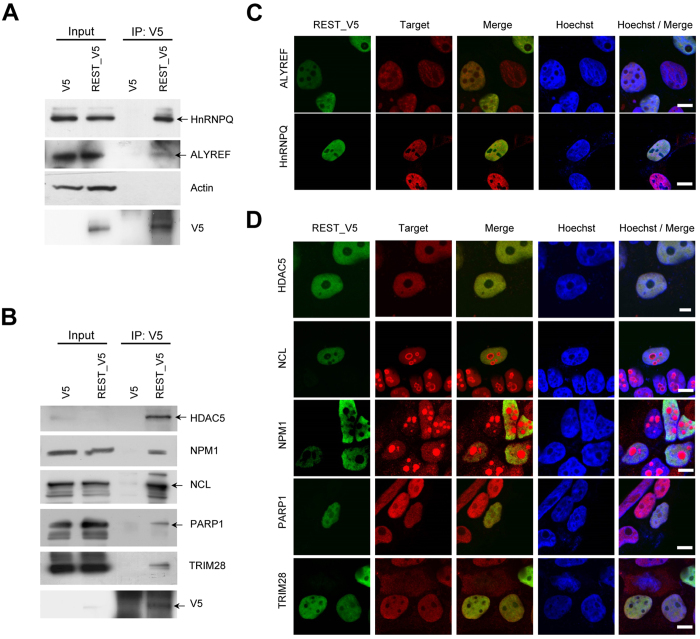
Validation of interaction and co-localization of candidate proteins interacting with REST. (**A**,**B**) Co-immunoprecipitation of interacting proteins with REST. Co-immunoprecipitation was performed by mixing V5 antibody-conjugated beads with cell lysates of HEK293FT cells transfected with pcDNA_V5 or pcDNA_REST-V5 vectors. mRNA processing/splicing associated target proteins including ALYREF and HnRNP Q (**A**) and transcription-related target proteins including HDAC5, NPM1, NCL, PARP1, and TRIM28 (**B**) were confirmed by co-immunoprecipitation coupled with western blotting. Arrows indicate the target proteins. Full-length blots are included in the Supplementary Figure 3. (**C**,**D**) Co-localization of REST with interacting proteins such as ALYREF, HnRNP Q (**C**), HDAC5, NPM1, NCL, PARP1, and TRIM28 (**D**). HEK293FT cells transfected with REST-V5 expressing vector were fixed after 24 h, followed by probing with the indicated target specific antibody and V5 antibody. Images analyzed at magnification of 400x. Scale bar indicates 10 μm.

We confirmed the associations between REST and the interacting proteins involved in mRNA processing/splicing and transcription using co-immunoprecipitation coupled with western blotting. Among the mRNA processing/splicing-related interacting proteins, we validated the interactions of ALYREF and HnRNPQ. These proteins were clearly co-immunoprecipitated with REST in REST-expressing HEK293FT cell lysates, whereas the interacting targets were not co-immunoprecipitated in control cell lysates (Fig. 3A). When actin was used to confirm the interaction with REST as a negative control, it was not co-immunoprecipitated with REST, supporting the reliability of our co-immunoprecipitation results (Fig. 3A). Immunocytochemistry further demonstrated that REST was co-localized with these two proteins in the nucleus (Fig. 3C).

We also validated the interaction of REST with novel interacting proteins associated with transcription. HDAC5 was co-immunoprecipitated, as previously reported^26^, as were other novel interacting proteins, such as NPM1, NCL, PARP1, and TRIM28 (Fig. 3B). The co-localizations of these REST-interacting proteins were further confirmed by immunocytochemistry, which showed that HDAC5, PARP1, and TRIM28 were clearly co-localized with REST in the nucleus (Fig. 3D), while NPM1 and NCL were also detected and merged with REST in the nucleoplasm, despite being predominantly localized in the nucleoli (Fig. 3D).

### Significant overlap of target genes regulated by TRIM28 and REST

**Figure 4:**
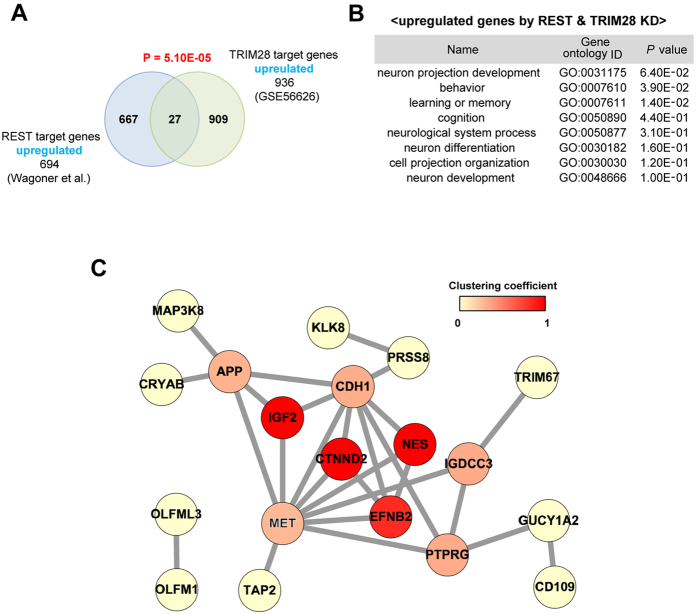
Co-regulated genes by REST and TRIM28. (**A**) The overlap between differentially expressed transcripts following knockdown of REST and REST-interacting proteins is depicted as Venn diagrams. Blue and green circles indicate transcripts regulated by REST and TRIM28, respectively. (**B**) Gene Ontology (GO) analysis using gene sets upregulated by REST and TRIM28 knockdown. (**C**) Network analysis of gene set upregulated by REST and TRIM28 knockdown. Edges were drawn based on the STRING databases. The node color indicates the clustering coefficency. Fisher’s test was used for statistical analysis.

Among the proteins in the interactome that were involved in transcription, TRIM28 was previously reported to be a transcriptional co-regulator and was implicated in multiple aspects of cellular physiology, including embryonic development^9^, similarly as REST. Thus, TRIM28 was subjected to further analysis to confirm its interaction with REST. We initially compared target genes whose transcription could be regulated by both TRIM28 and REST. We used a microarray dataset obtained using mouse embryonic stem cells collected before and after conditional TRIM28 fl/fl knockout^10^ to define TRIM28-regulated genes. Likewise, a list of genes that are upregulated by REST deficiency in three cell lines (HEK293, MCF10A, and T47D) was obtained using another public dataset^27^. There was a significant overlap in target genes that were upregulated by TRIM28 depletion and upregulated by REST knockdown (P = 5.1 × 10^−5^; Fig. 4A). To find significant overlap of target genes regulated by TRIM28 and REST, we additionally compared public datasets as obtained using the same cell type of mouse embryonic stem cells^28^. They performed systematic knockdown of 97 transcription factors including REST and TRIM28 with shRNA in mouse embryonic stem cells to decipher gene regulatory networks^28^. Significant overlap in target genes of TRIM28 and REST was also confirmed (P = 2.94 × 10^−179^; Supplementary Fig. 2).

Systematic analysis and biological experiments were further performed to determine the functional implications of REST and TRIM28 having shared target genes. We performed a Gene Ontology (GO) analysis to identify the functional links of the shared target genes (Supplementary Table 4) during biological processes. Interestingly, neuronal development-related GO terms such as “neuron projection development” and “regulation of neuronal differentiation” were significantly enriched in the gene sets that were upregulated by the knockdown of REST and TRIM28 (Fig. 4B). We also reconstructed a network model to identify central genes among the shared targets that were upregulated by REST and TRIM28 (Fig. 4C), which showed that adhesive junction-associated delta-catenin (CTNND2) was a central protein with a high clustering coefficient. Therefore, since CTNND2 has also been implicated in neuronal development^29^,^30^,^31^, it was subjected to further analysis.

### Expression levels of CTNND2, TRIM28, and REST during neuronal development

**Figure 5:**
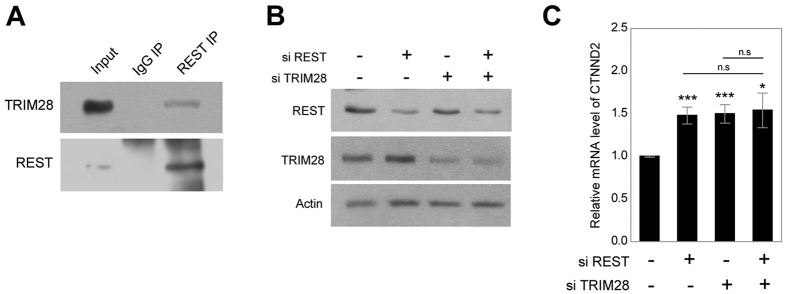
Expression of CTNND2 by REST and TRIM28 depletion. (**A**) Physical interaction between REST and TRIM28 in the mouse brain lysates. (**B**) SH-SY5Y cells transfected with an indicated siRNA. Levels of REST, TRIM28 and actin were assessed using western blot. Full-length blots are included in the Supplementary Figure 3. (**C**) mRNA levels of CTNND2 in SH-SY5Y cells transfected with the indicated siRNA are indicated in the histogram. Three independent experiments were performed, and the t-test was used for statistical analysis. (*P < 0.05; **P < 0.01; ***P < 0.001).

To investigate a physiological relevance of interactions of REST with TRIM28 in neuronal development, endogenous co-IP between these two proteins was performed using the mouse brain lysates. Interestingly, TRIM28 was co-immunoprecipitated with REST in adult mouse brain lysates, indicating that REST physiologically forms a complex with TRIM28 in the neurons (Fig. 5A). Next, we investigated the regulation of CTNND2 expression by REST and TRIM28. The siRNAs against REST and TRIM28 were transfected into SH-SY5Y cells, and significant reduction of REST and TRIM28 by siRNAs was confirmed by western blotting (Fig. 5B). Upon the depletion of REST or TRIM28, the mRNA levels of CTNND2 significantly increased (Fig. 5C). However, simultaneous knockdown of REST and TRIM28 did not show synergetic effects for the regulation of CTNND2 (Fig. 5C).

**Figure 6:**
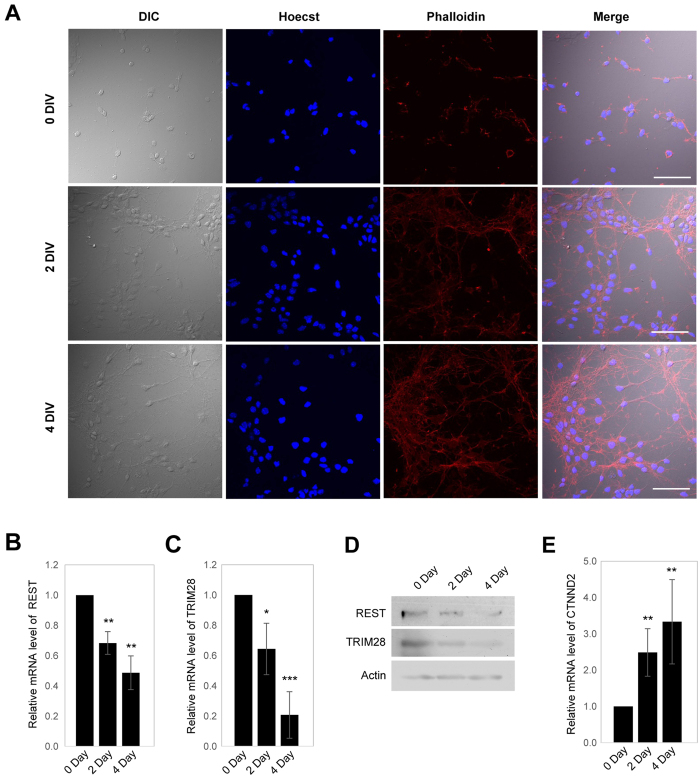
Expression of CTNND2, REST and TRIM28 during the differentiation of primary neuron. (**A**) Primary neurons were obtained from mouse embryo and maintained in the culture plate. The primary neurons were fixed on days 0, 2 and 4, followed by probing with Phalloidin. Scale bar indicates 50 μm. (**B**–**D**) mRNA levels of REST (**B**), TRIM28 (**C**) and protein levels of REST, TRIM28 (**D**) were accessed in the primary neurons on days 0, 2 and 4. (**E**) mRNA level of CTNND2 accessed in the primary neurons on days 0, 2 and 4. Three independent experiments were performed to analyze alteration of genes at mRNA level, and the t-test was used for statistical analysis. (*P < 0.05; **P < 0.01; ***P < 0.001).

To investigate the physiological relevance of the regulation of CTNND2 expression by REST and TRIM28, we assessed the expression levels of all three genes during neuronal differentiation. It has previously been shown that primary neurons in culture reflect the development of primary neurons *in vivo*^32^, and thus, primary neurons were obtained from an embryonic mouse brain and cultured *in vitro*. The filamentous actin (F-actin) in these neurons was stained with Phalloidin containing a fluorescent tag that specifically binds to F-actin to confirm neuronal differentiation on days 0, 2, and 4. A high density of F-actin was detected on day 4, indicating dendritic outgrowth (Fig. 6A). Expression levels of REST and TRIM28 were assessed in primary neurons obtained on days 0, 2, and 4 at both mRNA and protein levels. Interestingly, as the primary neurons differentiated, the expressions of REST and TRIM28 decreased at both mRNA and protein levels (Fig. 6B–D). On the other hand, expression of CTNND2 increased at the mRNA level during the course of day (Fig. 6E).

### Regulation of neurite outgrowth by REST and TRIM28

**Figure 7:**
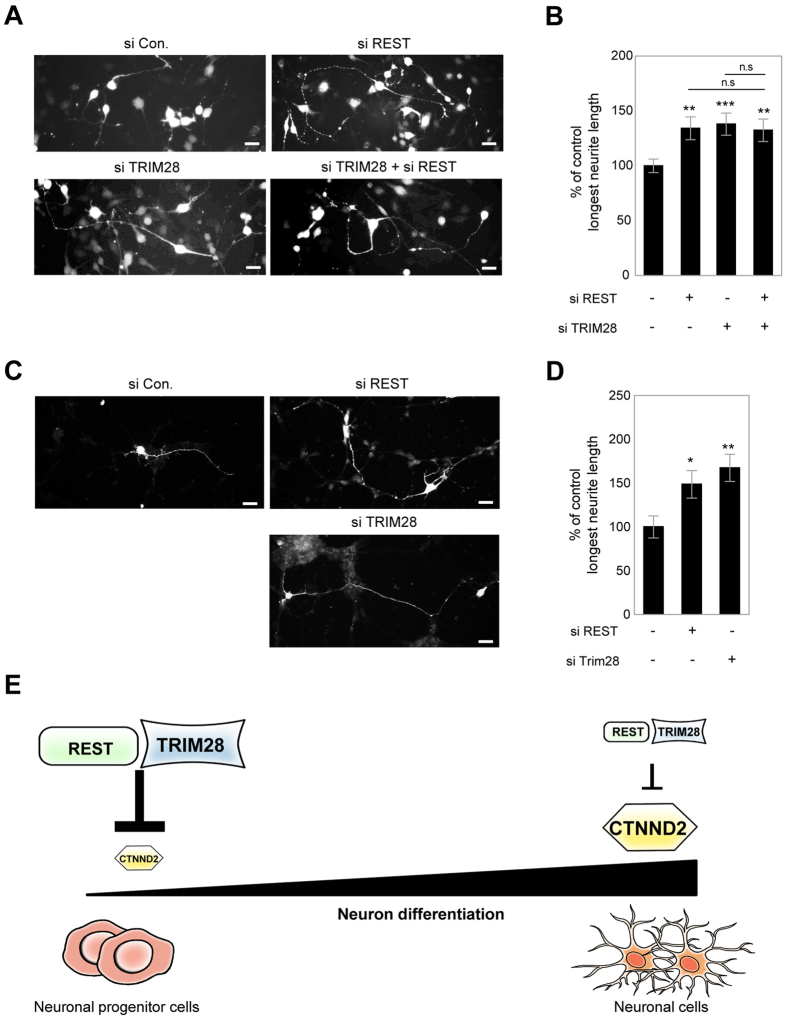
The regulation of neurite outgrowth by REST-TRIM28 complex and postulated model of role of REST and TRIM28 in the regulation of CTNND2 expression during neuron differentiation. (**A**) Representative images of differentiated CAD cells transfected with pmCherry-N1 and siRNAs. Scale bar indicates 30 μm. (**B**) The longest neurite length normalized to soma diameter in siRNA-mediated knockdown groups was normalized to that in the control group (n = 50 for control siRNA, 35 for REST siRNA, 33 for TRIM28 siRNA, 41 for REST siRNA + TRIM28 siRNA). (**C**) Representative images of cultured primary cortical neurons transfected with pmCherry-N1 and siRNAs. Scale bar indicates 20 μm. (**D**) The longest neurite length normalized to soma diameter in siRNA-mediated knockdown groups was normalized to that in the control group (n = 27 for control siRNA, 22 for REST siRNA, 19 for TRIM28 siRNA). The t-test was used for statistical analysis. (*P < 0.05; **P < 0.01; ***P < 0.001) (**E**) REST and TRIM28 might repress the expression levels of CTNND2 in neural progenitor cells. As the neural progenitor cells differentiate, levels of REST and TRIM28 decrease, which may induce expression of CTNND2 as a result of the loss of transcriptional suppression by REST and TRIM28.

Neurite outgrowth is a key process during neuronal migration and differentiation that are essentially required for neuronal development^33^. Notably, REST is involved in neurite outgrowth in differentiated neuroblastoma cell line and primary neurons^34^. On the other hand, the role of TRIM28 in neurite outgrowth is unclear although several evidences have suggested a potential link between TRIM28 and embryonic development^9^. Thus, we investigated whether TRIM28 could be involved in the regulation of neurite outgrowth like REST. Thus, FITC-conjugated siRNAs for mouse TRIM28 or REST were generated and knockdown efficiencies of siRNAs were confirmed by Western blot (Supplementary Fig. 1). Consistent with previous reports, siRNA-mediated depletion of REST resulted in the longest neurite outgrowth in Cath.a differentiated (CAD) mouse neuroblastoma cell line (Fig. 7A,B). Interestingly, the longest neurite length was also increased upon TRIM28 knockdown compared to control knockdown (Fig. 7A,B). Simultaneous knockdown of REST and TRIM28 did not show significant differences compared to single knockdown of REST or TRIM28 (Fig 7A,B). We also performed the neurite outgrowth assay in cultured primary cortical neurons. Similar to the results in CAD cell line, siRNA-mediated knockdown of REST or TRIM28 increased the longest neurite length compared to control knockdown, revealing the physiological relevance of the regulation of neurite outgrowth by REST and TRIM28 (Fig. 7C,D).

Figure 7E shows a proposed model of how REST and TRIM28 interact to regulate CTNND2 expression during neuronal differentiation. In the neuronal progenitor cells, REST and TRIM28 may be highly expressed to repress the expression of CTNND2. However, as the neuronal progenitor cells differentiate, the expression levels of REST and TRIM28 decrease, which may lead to an increase in CTNND2 as a result of the loss of transcriptional suppression by REST and TRIM28.

## Discussion

This systematic study investigated protein–protein interaction profiles for REST using AP–MS. The interactome revealed more than 200 new interactions between REST and proteins involved in mRNA processing/splicing, chromatin organization, and transcription. Immunoprecipitation and immunofluorescent staining confirmed the ability of REST to bind to some of these interacting proteins, and further analysis of the interaction between REST and TRIM28 implied that TRIM28 to be a corepressor of REST in the regulation of genes involved in neuronal development.

REST protein in HEK293 cell line was located at about 200 kD on the gel, which is greater than the predicted molecular weight estimated by its sequence (approximately 116 kD). Consistent with our data, the size of REST has been reported to be about 200 kD in various mammalian cell lines including HEK293 cell line^11^,^35^. The greater size of REST than its estimated molecular weight could be explained by post-translational modification. O-linked glycosylation has been reported as the major cause for the shift of molecular weight of proteins^36^,^37^. REST4, an isoform of REST, was reported to be O-glycosylated between the residues at 87 and 152^38^. Thus, the detection of about 200 kD REST might be attributed to its glycosylation, but direct mapping of glycosylation site on full length of REST warrants for investigation.

REST-interacting proteins that have been identified previously are summarized in Supplementary Table 5, each of which was independently identified or confirmed by yeast two-hybrid screening or co-immunoprecipitation under different experimental conditions. We firstly successfully identified over 200 REST-interacting proteins using AP–MS under physiological conditions in a relatively unbiased manner as a result of advancements in mass spectrometry^7^. Our interactome not only included novel binding partners but also confirmed REST-interacting proteins that had been identified previously, such as HDAC5, which has been shown to be recruited to the N-terminus of REST^26^. Though only one previously identified interacting partner was included in our intractome due to the limited number of known REST-interacting proteins, it is statistically significant. The reason why well-established interacting proteins such as Sin3A, HDAC1^39^,^40^ and HDAC2^34^ were not detected in our analysis can be due to the fact that AP-MS could depend on the expression levels of proteins in host cell which we used to make cell lysate. Although CoREST, mSin3 and HDAC1/2 were expressed in HEK293T cell based on microarray data of HEK293T cell (One of ENCODE transcriptome data^41^; GEO accession: GSE15805^42^), their expression was not that high. In addition, CoREST is known to be negatively co-expressed with REST from SEEK database^43^. Thus, endogenous expression level of each protein in HEK293 cells should affect the detection of REST-interacting proteins. Our interactome mainly included nuclear and cytoplasmic proteins, which is consistent with previous reports that have identified the nuclear and cytoplasmic localization of REST^44^,^45^,^46^. Although immunofluorescence staining showed that REST is mainly localized in the nucleus (Fig. 1B), endogenous REST has been detected in the cytoplasmic and nuclear fractions of neural cell lines, primary neurons, and adult mouse brain tissues^44^,^45^. It has previously been shown that REST can be translocated into the nucleus by binding to REST/NRSF-interacting LIM domain protein (RILP) in the nuclear membrane, and it has been observed in the cytosol following RILP mutation^46^. The enrichment of cytoplasmic proteins in the REST interactome could also be explained by the fact that many cytoplasmic proteins occupy a higher fraction of the whole proteome when analyzed using mass spectrometry^47^. Thus, our interactome dataset included a wide range of novel REST-interacting proteins under physiological conditions, most of which were nuclear or cytoplasmic proteins.

Proteins involved in mRNA processing/splicing were newly identified as REST-interacting proteins. We validated the interaction and co-localization of REST with ALYREF and HnRNP Q, which are involved in mRNA processing/splicing, using co-immunoprecipitation and immunofluorescent staining (Fig. 3A,C). ALYREF, an RNA-binding protein, is known to be localized to the nuclear speckle and to promote the export of matured mRNA as one of export factors^48^. HnRNP Q is a member of the cellular heterogeneous nuclear ribonucleoprotein (HnRNP) family, which regulates the packaging of nascent transcripts, alternative splicing, polyadenylation, and other aspects of mRNA metabolism and transport by binding to RNA^49^. The REST interactome also included eight additional HnRNPs (HnRNP M, HnRNP U, HnRNP R, HnRNP A0, HnRNP D0, HnRNP A3, HnRNP L, and HnRNP F). The functional role of the interaction between REST and ALYREF or HnRNPs in mRNA processing/splicing remains to be investigated. However, the enrichment of proteins associated with mRNA processing/splicing in our interactome implies that REST has a crucial role in cellular processes involving mRNA.

Since REST regulates transcription in the chromatin region, it was expected to interact with histone proteins involved in chromatin organization and transcription factors. Supporting this, our interactome included a wide range of novel interacting proteins that are involved in these processes. Several of the REST-interacting protein candidates with roles in transcription were subjected to verification tests, which confirmed that HDAC5, NPM1, NCL, PARP1, and TRIM28 clearly interacted and were co-localized with REST (Fig. 3B,D). Among these proteins, NPM1 and NCL are important nucleolar phosphoproteins with pleiotropic functions not only in transcription but also in various cellular processes, such as ribosome biogenesis, RNA metabolism, chromatin structure, rDNA transcription, and cell proliferation and growth^50^,^51^. NPM1 has been shown to function as an AP2a-binding transcriptional corepressor during cell differentiation^52^, while NCL has been reported to repress the alpha-1 acid glycoprotein gene^53^ and to affect the transcriptional activity of Myb transcription factor^54^. Interestingly, it has also been suggested that NCL interplays with REST at the overlapping REST/NCL binding sites within the promoters of CD59 and mcl1^55^, which may indirectly support our data showing an interaction between NCL and REST. PARP1 has also been linked to transcriptional regulation as a promoter-specific coactivator or corepressor for a number of different sequence-specific DNA-binding transcriptional regulators, including NF-ĸB, HES1, B-Myb, Oct-1, and NFAT^56^. HMGA1 is a chromatin remodeling protein that can regulate transcription and is present at high levels in undifferentiated tissues but at much lower levels in adult tissues^57^; this matches the expression pattern of REST during development^2^,^4^. Therefore, although the involvement of REST in mediating transcriptional regulation by HMGA1 has not yet been studied, the similar expression patterns of these two proteins during development and their interaction may imply a functional association between them. Taken together, our interactome included many new proteins that are involved in transcriptional regulation, which is consistent with the major function of REST.

It is possible that REST and TRIM28 may co-regulate CTNND2, which is involved in neuronal development. TRIM28 is mainly known as a corepressor in many repression complexes^8^. A comparison of the transcripts that were upregulated by depletion of REST or TRIM28 indicated a significant overlap (Fig. 4A). There was a significant overlap of upregulated genes in NT2-D1 cells upon the knockdown of REST or RNF2, one of the components of polycomb repressive complexes (PRCs), which are known to interact with REST and exist on REST binding sites^6^. Interestingly, it has also been reported that TRIM28 binds with PRC1 to suppress differentiation-inducible genes^10^. The sharing of a binding partner, such as PRC, may support the possibility of an interaction between REST and TRIM28. TRIM28 has been implicated in multiple aspects of cellular physiology, including embryonic development^9^,^10^, and the shared target genes that were upregulated following the knockdown of REST and TRIM28 were found to be associated with neuronal development (Fig. 4B). Among these co-regulated genes, CTNND2 exhibited the highest coefficient. CTNND2 was found to be upregulated upon the depletion of REST and TRIM28 in SH-SY5Y (Fig. 5C) and also increased following a reduction in REST and TRIM28 during primary neuronal differentiation (Fig. 6E), indicating the physiological relevance of the co-regulation of CTNND2 by REST and TRIM28. However, synergetic effect of simultaneous knockdown of REST and TRIM28 was not observed, indicating that they might complementarily repress the transcription of CTNND2 gene. CTNND2, which is a component of the cadherin–catenin cell adhesion complex, is an important neurodevelopmental protein due to its involvement in the regulation of spine and synapse morphogenesis, which is important for neuronal circuit formation and function^29^,^30^, and its loss has been associated with autism and strongly related to neurodevelopmental disorders^29^. We analyzed the neurite outgrowth, a crucial step of neuronal development, in REST- or TRIM28-knockdowned CAD cell line or primary cultured neurons, and could assess the contribution of REST and TRIM28 to the regulation of neurite outgrowth. Our results suggest that REST-TRIM28 complex might play essential roles in neuronal development by co-regulating the expression of CTNND2 gene. Thus, the correct induction of CTNND2 expression through the reduction of REST and TRIM28 may be crucial for normal neuronal development and the prevention of autism, and TRIM28 may be involved in the regulation of CTNND2 by acting as a corepressor of REST.

## Conclusions

Our interactome dataset included a wide range of novel REST-interacting proteins under physiological conditions, most of which were nuclear or cytoplasmic proteins. The enrichment of proteins associated with mRNA processing/splicing, chromatin assembly and transcriptional regulation in the interactome implies that REST has significant roles in these cellular processes. Among the target genes that were co-regulated by REST and TRIM28, we confirmed that CTNND2 was upregulated following the knockdown of REST or TRIM28. During differentiation of the primary neurons, levels of REST and TRIM28 gradually decreased, while that of CTNND2 increased. In addition, neurite outgrowth was increased by knockdown of REST or TRIM28 expressions, suggesting that reduction of REST and TRIM28 expressions could promote neuronal differentiation via activation of CTNND2 expression. Thus, this interactomic study of REST revealed novel interacting proteins that may represent a valuable resource for further investigating and also suggested functional links between REST and TRIM28 during neuronal development.

## Methods

### Cell culture, mouse lines, plasmids and siRNA

HEK293FT (Invitrogen), SH-SY5Y (Sigma) and CAD (Sigma) cells were cultured at 37 °C under 5% CO_2_ in DMEM supplemented with 10% FBS, 100 units/ml of penicillin and 100 μg/ml streptomycin. Mouse embryonic cortical neurons were cultured in the Neurobasal medium (GIBCO) supplemented with B27, glutamine and penicillin/streptomycin. B57BL/6 mice were purchased from Hyochang Science and embryos were processed for the culture of primary neurons. All animal procedures were approved by the Pohang University of Science and Technology Institutional Animal Care and Use Committee. All experiments were carried out in accordance with the approved guidelines.

Lipofectamine 2000 (Invitrogen) was used to transfect expression vectors and siRNAs into cells. The same control and REST expression vectors were used as described previously^11^,^16^. A mixture of four kinds of siRNAs targeting TRIM28 or REST was purchased from Dharmacon (USA). A mixture of eight kinds of FITC-conjugated siRNAs against mouse TRIM28 and REST was purchased from Bioneer (Korea). Sequences are shown in Supplementary Table 6.

### Co-immunoprecipitation of potential REST-interacting proteins and western blotting

Cells were harvested 24 h after transfection in cold PBS and centrifuged at 800 *g*. Cells were lysed with a lysis buffer containing 50 mM Tris-HCl, pH 7.4, 150 mM NaCl, 5 mM EDTA, 0.2% TritonX-100, 15 U/ml DNAse I and the inhibitor cocktail of proteases and phosphateases (Complete ULTRA Tablet and PhosSTOP, Roche) followed by the activation of DNases at room temperature for 10 min. REST and its interacting proteins were pulled down with anti-V5 antibody immobilized on agarose beads (Sigma), and then subjected to mass spectrometric analysis or western blot. For co-immunoprecipitation in adult mouse brain lysates, whole brain tissues from B57BL/6 mouse were homogenized and sonicated in same lysis buffer described above, and precleared by centrifugation for 10 min at 12,000 × g. The supernatants were incubated with IgG or anti-REST antibody (Abcam) overnight at 4 °C, and then incubated with protein A-agarose beads (GE-Healthcare) for 2 h at 4 °C.

All samples boiled at 100 °C for 5 min were subjected to SDS-PAGE and then transferred onto PVDF membranes (Millipore, USA). After treatment with 5% skimmed milk in TBS-T buffer (50 mM Tris-HCl, pH 7.4, 150 mM NaCl, 0.1% Tween20) for 1 h, membranes were incubated with the primary antibodies at 4 °C with shaking overnight. After incubated with the secondary antibody for 2 h, immunocomplexes were detected by use of the femto, Pico or ECL reagents (Thermo).

Antibodies for immunoprecipitation and western blot were from the companies as follows; rabbit anti-REST antibodies (Millipore, Abcam), mouse anti-V5 antibody (Sigma), mouse ant-ALY antibody (Santa Cruz), mouse anti-HnRNP Q (Santa Cruz), anti-HDAC5 (Santa Cruz), rabbit anti-B23 antibody (Santa Cruz), rabbit anti-C23 antibody (Santa Cruz), rabbit anti-PARP1 antibody (Cell signaling) and rabbit and mouse anti-TRIM28 antibodies (Santa Cruz, Abcam).

### In-solution digestion

Proteins were digested with trypsin as described previously with minor modifications^58^. Briefly, eluted proteins were lyophilized and resolved in a digestion solution of 6 M urea and 40 mM ammonium bicarbonate in high-performance liquid chromatography (HPLC)-grade water. Protein reduction was performed with 5 mM Tris (2-arboxyethyl) phosphine hydrochloride for 1 h, followed by alkylation with 25 mM iodoacetamide in the dark for 30 min at room temperature. The sample was in-solution digested with 5 ng/mL sequencing-grade modified trypsin (Promega, USA) for 16 h at 37 °C.

### Nano-LC-MS/MS

Peptides were analyzed using mass spectrometry as described previously with some modifications^58^. Tryptic peptides from in-gel digestion were separated using a homemade microcapillary column (75 μm × 12 cm) packed with C18 resin (Michrom Bioresources, USA). Samples were eluted using a linear gradient of a mixture of solvents A (0.1% formic acid in 2% acetonitrile) and B (0.1% formic acid in 98% acetonitrile), where the percentage of the latter mobile phase increased over 120 min at a flow rate of 0.26 μL/min: 2–50% over 94 min, 50–90% over 6 min, 90% over 6 min, 90–2% over 6 min, and 2% over 8 min. Eluted peptides were analyzed with an LTQ Velos Orbitrap mass spectrometer (Thermo Finnigan, USA) equipped with nano-ESI. MS precursor ion scans were acquired within a m/z range between 150 and 2000. The five most abundant ions detected in the precursor MS scan were dynamically selected for MS/MS analyses. Collision-induced dissociations of the selected precursor ions were performed in an ion trap (LTQ) with 35% normalized collision energy. We employed dynamic exclusion to increase the size of proteome to be detected as follows: repeat count for dynamic exclusion = 1, repeat duration = 30 s, dynamic exclusion duration = 180 s, and list size of dynamic exclusion = 50.

### Identification and quantification of proteins

Peak lists of MS data were generated, and identification/quantification of peptides and proteins from three technical replicates of LC-MS/MS data performed using the MaxQuant quantification tool with Andromeda search engine (version 1.3.0.5)^59^,^60^,^61^. The top 10 peaks per 100 Da were used for analysis. Enzyme specificity for trypsin was used. The minimal peptide length was six amino acids, and two mis-cleavages were allowed. Variable modification options were employed for oxidation of methionine (15.995 Da) and carbamidomethylation of cysteine (57.021 Da). Tolerance was set to 10 ppm for precursor ions and 0.8 Da for fragment ions. Swiss-Prot database (Homo sapiens reference proteome set, release 2013_01, 20,226 entries) with added contaminants and reverse sequences was used. For peptide and protein identification, 1% false discovery rate (FDR) was determined by accumulating 1% of reverse database hits. Common peptides shared by two proteins were combined and reported as one protein group. The first majority protein ID was selected as the representative protein of each group, and used as protein ID for further analysis. For comparison of samples, we used label-free quantification with a minimum of two ratio counts to determine the normalized protein label-free quantification (LFQ) intensity^62^. Raw proteome data have been deposited in the ProteomeXchange Consortium (http://proteomecentral.proteomexchange.org) via the PRIDE partner repository with the dataset identifier PXD003210.

### Definition of REST-interacting proteins

For analysis of comparative proteomics, proteins with more than two unique peptides and lower than 0.01 posterior error probability were selected. We additionally removed all the reverse sequences and contaminant proteins. Using LFQ intensity, fold change was calculated with the equation described in a previous report^63^. REST-binding proteins were identified as those enriched in the REST-positive sets, respectively, displaying more than 0.5-fold change.

### Subcellular localization of proteins

To analyze subcellular localization of the REST interactome, we extracted the related information from UniProt (Release 2013_01). P-values for localization of proteins compared with the whole proteome were assessed by Fisher’s test.

### Systematic analysis

DAVID 6.7^64^ was employed for Gene Ontology analysis. Biological Process terms of Gene Ontology were used, and P-values were calculated with modified Fisher’s exact test^65^. The protein-protein interaction database from the NCBI Entrez Gene^66^, updated on Feb 17 in 2013, was applied to build the hypothetical network, which was visualized with Cytoscape 2.8.3^67^.

### Microarray data

A published gene expression dataset (GSE56626 for gene set regulated by TRIM28, GSE26520 for gene sets regulated by TRIM28 or REST) was used to compare gene sets upregulated by REST^27^. The raw data were retrieved from the gene expression omnibus public gene expression database. Target genes upregulated by REST and TRIM28 were compared using Venny 2.1, and P-values were calculated with Fisher’s exact test.

### Immunocytochemical staining

HEK293FT cells or hippocampal neurons were seeded on cover slips coated with poly-D-lysine, fixed with 4% paraformaldehyde in PBS, permeabilized by incubation with 0.1% Triton X-100 in PBS, and subsequently blocked with 1% BSA in PBS for 2 h. To verify the cellular localization of REST expressed ectopically, the HEK293FT cells were incubated with mouse anti-V5 antibody (Life technology) or rabbit anti-REST antibody (Millipore) in the cold room overnight. To confirm the co-localization of REST-interacting proteins and REST, the HEK293FT cells were incubated together with the following antibodies: goat anti-HDAC5 antibody (Santa Cruz), rabbit anti-TIF1ß antibody (Santa Cruz), mouse anti-ALYREF (Santa Cruz), mouse anti-HnRNP Q (Santa Cruz), mouse anti-NPM1 (Santa Cruz), mouse anti-NCL (Santa Cruz) and rabbit anti-PARP1 (Cell signaling). The cells were washed with PBS for three times and incubated with anti-mouse Alexa Fluor 488 (Invitrogen) and anti-rabbit Alexa Fluor 594/anti-goat Alexa Fluor 594 (Invitrogen) for 1 h at room temperature in the dark. To confirm the expression of F-actin, the hippocampal neurons were incubated with Alexa Fluor 568 Phalloidin (Thermo) for 1 h at room temperature in the dark. The HEK293FT cells and hippocampal neurons were subsequently washed with PBS, incubated with Hoechst 33342 (Invitrogen) for 10 min at room temperature, and mounted with Mounting medium (Dako).

### RT-PCR

Total RNA was isolated using the RNeasy mini Kit (Qiagen) according to the manufacture instructions and then reverse-transcribed to cDNA (BioRad). Synthesized cDNA was used as the template for real-time quantitative PCR analysis with target gene-specific primers. Primer sequences are shown in Supplementary Tables 7 and 8.

### Neurite outgrowth assay

CAD cells were transfected with pmCherry-N1 (Clontech) and FITC-conjugated siRNAs targeting mouse REST or TRIM28 using Lipofectamine 2000 as described previously^68^. At 48 h post transfection, cells were detached and seeded again onto plates and then cultured for 48 h in serum-free DMEM media for differentiation. After the cells were fixed, fluorescent cells were randomly selected by fluorescent microscopy (X20 objective). Neurite length and the diameter of soma were measured by Image J software (National Institute of Health), and the length of the longest neurite was normalized to soma diameter. For cultured mouse primary neurons, neurons were transfected with pmCherry-N1 and siRNAs by Lipofectamine 2000 at 15 h post seeding and cultured for additional 3 days. Neurons were fixed and randomly selected to measure neurite length and the diameter of soma as above. The longest neurite length was normalized to soma diameter.

## Additional Information

**How to cite this article**: Lee, N. *et al*. Interactomic analysis of REST/NRSF and implications of its functional links with the transcription suppressor TRIM28 during neuronal differentiation. *Sci. Rep.* **6**, 39049; doi: 10.1038/srep39049 (2016).

**Publisher's note:** Springer Nature remains neutral with regard to jurisdictional claims in published maps and institutional affiliations.

## Supplementary Material

Supplementary Information

Supplementary Tables
