# Supplementary material for: Interactomic analysis of REST/NRSF and implications of its functional links with the transcription suppressor TRIM28 during neuronal differentiation
Source: Sci Rep. 2016 Dec 15;6:39049. doi: 10.1038/srep39049 (PMC5157023; doi:10.1038/srep39049)
Supplement: Supplementary Information [file srep39049-s1.pdf]

Interactomic analysis of REST/NRSF and implications of its functional links with the transcription suppressor TRIM28 during neuronal differentiation.
; Namgyu Lee*, Sung Jin Park*, Dae-Kyum Kim, Ghazal Haddad, Seon-Min Park, Sang Ki Park, Kwan Yong Choi

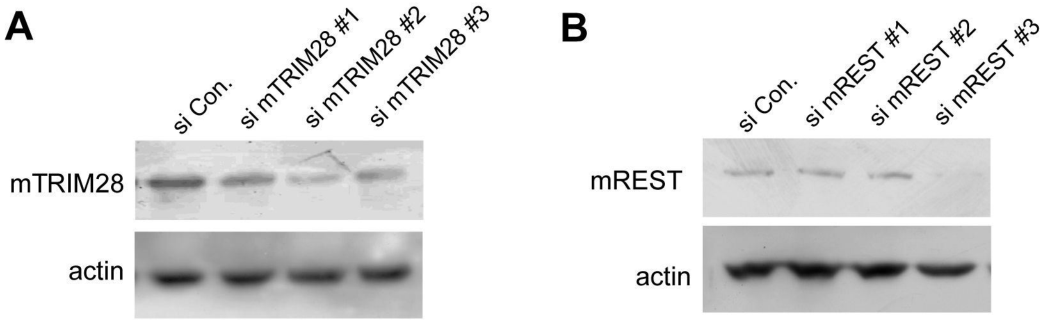

Sup. Fig. 1

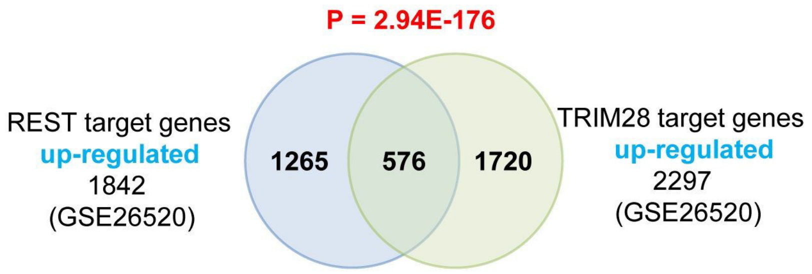
P = 2.94E-176
REST target genes
up-regulated
1842
(GSE26520)
1265
576
1720
TRIM28 target genes
up-regulated
2297
(GSE26520)

Sup. Fig. 2

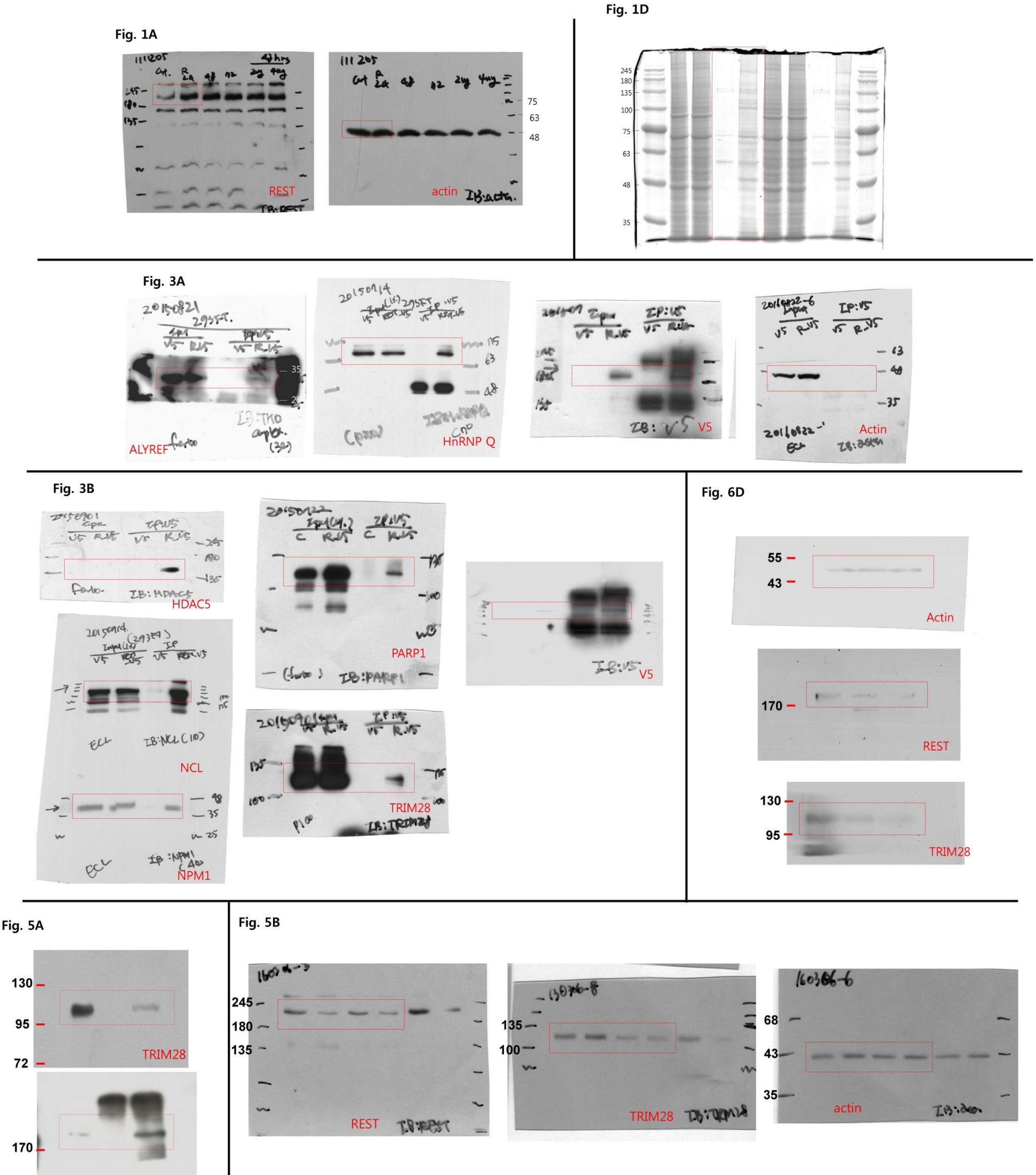
Fig. 1A
REST
actin
Fig. 1D
Fig. 3A
ALYREF
HnRNP Q
V5
Actin
Fig. 3B
HDAC5
PARP1
V5
NCL
TRIM28
NPM1
Fig. 6D
Actin
REST
TRIM28
Fig. 5A
TRIM28
REST
Fig. 5B
REST
TRIM28
actin

Sup. Fig. 3

Interactomic analysis of REST/NRSF and implications of its functional links with the transcription suppressor TRIM28 during neuronal differentiation

; Namgyu Lee, Sung Jin Park, Dae-Kyum Kim, Ghazal Haddad, Seon-Min Park, Sang Ki Park, Kwan Yong Choi

Supplementary Table 5. List of REST-interacting proteins identified previously

Interacting proteins	Experiment	Reference
mSin3	pull down	[1]
Sin3A	co-IP	[2]
CoREST	pull down, yeast two-hybrid screening	[3]
G9a	co-IP	[4]
CtBP	co-IP	[5]
BRG1 (SMARCA4)	co-IP	[6]
HDAC1	co-IP	[2]
HDAC2	co-IP	[7]
HDAC4	co-IP	[8]
**HDAC5**	co-IP	[8]
MeCP2	ChIP	[9]
Zfp90	yeast two hybridization, co-IP	[10]
PC4	co-IP, pull down	[11]
SWI/SNF complex; BRG1, Brm, BAF155	Co-IP	[12]
RILP	yeast two hybridization, co-IP	[13]
Kaposin A (KSHV latency, in cytoplasm)	co-IP	[14]
Huntingtin	co-IP	[15]
ß-TRCP	pull down	[16]
HAUSP	co-IP, immucocytochemistry	[17]
TBP	co-IP	[18]
Polycomb Repressor Complex 1 (PRC1) and PRC2; Suz12, Ezh2, Eed, Rnf2, Nspc1	co-IP	[19]
TSPYL2	Mass spectrometry, Co-IP	[20]
TRF2	co-IP, immucocytochemistry	[21]

* Bold is detected in our interactome.

References

1. Nomura M, Uda-Tochio H, Murai K, Mori N and Nishimura Y. The neural repressor NRSF/REST binds the PAH1 domain of the Sin3 corepressor by using its distinct short hydrophobic helix. J Mol Biol. 2005; 354(4):903-915.
2. Huang Y, Myers SJ and Dingledine R. Transcriptional repression by REST: recruitment of Sin3A and histone deacetylase to neuronal genes. Nat Neurosci. 1999; 2(10):867-872.
3. Andres ME, Burger C, Peral-Rubio MJ, Battaglioli E, Anderson ME, Grimes J, Dallman J, Ballas N and Mandel G. CoREST: a functional corepressor required for regulation of neural-specific gene expression. Proceedings of the National Academy of Sciences of the United States of America. 1999; 96(17):9873-9878.
4. Roopra A, Qazi R, Schoenike B, Daley TJ and Morrison JF. Localized domains of G9a-mediated histone methylation are required for silencing of neuronal genes. Molecular cell. 2004; 14(6):727-738.
5. Garriga-Canut M, Schoenike B, Qazi R, Bergendahl K, Daley TJ, Pfender RM, Morrison JF, Ockuly J, Stafstrom C, Sutula T and Roopra A. 2-Deoxy-D-glucose reduces epilepsy progression by NRSF-CtBP-dependent metabolic regulation of chromatin structure. Nat Neurosci. 2006; 9(11):1382-1387.
6. Ooi L, Belyaev ND, Miyake K, Wood IC and Buckley NJ. BRG1 chromatin remodeling activity is required for efficient chromatin binding by repressor element 1-silencing transcription factor (REST) and facilitates REST-mediated repression. The Journal of biological chemistry. 2006; 281(51):38974-38980.
7. Roopra A, Sharling L, Wood IC, Briggs T, Bachfischer U, Paquette AJ and Buckley NJ. Transcriptional repression by neuron-restrictive silencer factor is mediated via the Sin3-histone deacetylase complex. Molecular and cellular biology. 2000; 20(6):2147-2157.
8. Nakagawa Y, Kuwahara K, Harada M, Takahashi N, Yasuno S, Adachi Y, Kawakami R, Nakanishi M, Tanimoto K, Usami S, Kinoshita H, Saito Y and Nakao K. Class II HDACs mediate CaMK-dependent signaling to NRSF in ventricular myocytes. J Mol Cell Cardiol. 2006; 41(6):1010-1022.
9. Ching GY and Liem RK. RE1 silencing transcription factor is involved in regulating neuron-specific expression of alpha-internexin and neurofilament genes. Journal of neurochemistry. 2009; 109(6):1610-1623.
10. Hata L, Murakami M, Kuwahara K, Nakagawa Y, Kinoshita H, Usami S, Yasuno S, Fujiwara M, Kuwabara Y, Minami T, Yamada Y, Yamada C, Nakao K, Ueshima K, Nishikimi T and Nakao K. Zinc-finger protein 90 negatively regulates neuron-restrictive silencer factor-mediated transcriptional repression of fetal cardiac genes. J Mol Cell Cardiol. 2011; 50(6):972-981.
11. Das C, Gadad SS and Kundu TK. Human positive coactivator 4 controls heterochromatinization and silencing of neural gene expression by interacting with REST/NRSF and CoREST. J Mol Biol. 2010; 397(1):1-12.
12. Watanabe H, Mizutani T, Haraguchi T, Yamamichi N, Minoguchi S, Yamamichi-Nishina M, Mori N, Kameda T, Sugiyama T and Iba H. SWI/SNF complex is essential for NRSF-mediated suppression of neuronal genes in human nonsmall cell lung carcinoma cell lines. Oncogene. 2006; 25(3):470-479.
13. Shimojo M and Hersh LB. REST/NRSF-interacting LIM domain protein, a putative nuclear translocation receptor. Molecular and cellular biology. 2003; 23(24):9025-9031.
14. Valiya Veettil M, Dutta D, Bottero V, Bandyopadhyay C, Gjyshi O, Sharma-Walia N, Dutta S and Chandran B. Glutamate secretion and metabotropic glutamate receptor 1

expression during Kaposi's sarcoma-associated herpesvirus infection promotes cell proliferation. PLoS Pathog. 2014; 10(10):e1004389.
15. Zuccato C, Tartari M, Crotti A, Goffredo D, Valenza M, Conti L, Cataudella T, Leavitt BR, Hayden MR, Timmusk T, Rigamonti D and Cattaneo E. Huntingtin interacts with REST/NRSF to modulate the transcription of NRSE-controlled neuronal genes. Nat Genet. 2003; 35(1):76-83.
16. Westbrook TF, Hu G, Ang XL, Mulligan P, Pavlova NN, Liang A, Leng Y, Maehr R, Shi Y, Harper JW and Elledge SJ. SCFbeta-TRCP controls oncogenic transformation and neural differentiation through REST degradation. Nature. 2008; 452(7185):370-374.
17. Huang Z, Wu Q, Guryanova OA, Cheng L, Shou W, Rich JN and Bao S. Deubiquitylase HAUSP stabilizes REST and promotes maintenance of neural progenitor cells. Nature cell biology. 2011; 13(2):142-152.
18. Murai K, Naruse Y, Shaul Y, Agata Y and Mori N. Direct interaction of NRSF with TBP: chromatin reorganization and core promoter repression for neuron-specific gene transcription. Nucleic acids research. 2004; 32(10):3180-3189.
19. Dietrich N, Lerdrup M, Landt E, Agrawal-Singh S, Bak M, Tommerup N, Rappsilber J, Sodersten E and Hansen K. REST-mediated recruitment of polycomb repressor complexes in mammalian cells. PLoS Genet. 2012; 8(3):e1002494.
20. Epping MT, Lunardi A, Nachmani D, Castillo-Martin M, Thin TH, Cordon-Cardo C and Pandolfi PP. TSPYL2 is an essential component of the REST/NRSF transcriptional complex for TGFbeta signaling activation. Cell Death Differ. 2015; 22(8):1353-1362.
21. Zhang P, Pazin MJ, Schwartz CM, Becker KG, Wersto RP, Dilley CM and Mattson MP. Nontelomeric TRF2-REST interaction modulates neuronal gene silencing and fate of tumor and stem cells. Curr Biol. 2008; 18(19):1489-1494.

Interactomic analysis of REST/NRSF and implications of its functional links with the transcription suppressor TRIM28 during neuronal differentiation

; Namgyu Lee, Sung Jin Park, Dae-Kyum Kim, Ghazal Haddad, Seon-Min Park, Sang Ki Park, Kwan Yong Choi

Supplementary Table 6. siRNA sequences targeting REST and TRIM28

siGENOME SMART pool, Human REST	D-006466-06, CGACAUGUAUGACUUGCAU
	D-006466-07, GGGCCUAAACCUCUUAAUU
	D-006466-08, GAUGGAGGGUGCCCAGAUA
	D-006466-09, CAGUAUAGUUUGUGAAAUG
siGENOME SMART pool, Human TRIM28	J-005046-07, GAAAUGUGAGCGUGUACUG
	J-005046-08, GCGAUCUGGUUAUGUGCAA
	J-005046-09, AGACAGCACUGGCGUGGUG
	J-005046-10, GAACGAGGCCUUCGGUGAC
siGENOME SMART pool, Mouse REST	siRNA#1 GUGAUCAGUGCAAUUAUGU
	siRNA#2 GAGAACGAGCGCAUCUACA
	siRNA#3 CAGAUAGAAGCAACUUCAA
siGENOME SMART pool, Mouse TRIM28	siRNA#1 CUCACAAGGACCAUCAGUA
	siRNA#2 CCACCAGUCUUCAAGGUCU
	siRNA#3 CAGCAUUGCUACUCUGGAU

Supplementary Table 7. Real-time quantitative PCR primers for human genes

Genes		Sequences
CTNND2	F	CCGTGTTTCCTCATCTATGG
	R	CTGCTGAATGCCTTGTTTAGT
GAPDH	F	CCTGGTATGACAACGAATTTGGC
	R	GTACATGACAAGGTGCGGCTC

Supplementary Table 8. Real-time quantitative PCR primers for mouse genes.

Genes		Sequences
CTNND2	F	GAAGTTGGTCGGCATCTCTA
	R	TTTGTCGATCCCTCTCGATG
REST	F	ATGGAAGTGACCTGAGTGAC
	R	GCGATTGAGGTGTTTGCTAT
TRIM28	F	GGAAATGTGAGCGTGTTCTC
	R	TGAACTGTTTGAACATGCGG
GAPDH	F	ACCTGCCAAGTATGATGACA
	R	GCCGTATTCATTGTCATACCAG
